# Parent satisfaction with a pediatric practice in Germany: A questionnaire-based study

**DOI:** 10.1186/1824-7288-37-31

**Published:** 2011-07-05

**Authors:** Anne Weissenstein, Alexandra Straeter, Gloria Villalon, Elisabeth Luchter, Stefan Bittmann

**Affiliations:** 1 Ped Mind Institute, Aerzte- und Finanzzentrum Epe, Hindenburgring 4, 48599 Gronau, Germany

**Keywords:** parent satisfaction, pediatrics, pediatric practice

## Abstract

**Background:**

Parental satisfaction with a pediatric day center is essential for the medical treatment of children, since it is closely related to compliance. In order to ascertain factors which predict parental satisfaction as well as to discover possible weak points, we developed a questionnaire.

**Methods:**

127 parents visiting the pediatric day center from October to November 2010 were asked to respond to a questionnaire. The survey was given to them directly by the doctor after their visit and it provided the opportunity to determine subjective and soft factors in quality management, which is essential for a pediatric practice. The questionnaire consisted of 27 items divided into three scales. The scales were as follows: satisfaction concerning the infrastructure and organization, satisfaction concerning the communicative and empathic competence of the doctor as well as the other staff, and finally the results and the overall impression. Moreover, the survey asked the respondents for their comments on the pediatric day center and sociodemographic data were queried.

**Results:**

A total of 67 parents (52,7%) responded to the survey. The mean parental satisfaction concerning infrastructure and organization achieved 3,61 (scale 1-very unsatisfied-through 4-very satisfied). The mean satisfaction with the expertise of the doctor and the staff was 3,56 and the overall satisfaction was 3,65. Ninety-one percent of the parents would visit the pediatric practice again and 84,2% would definitely recommend the practice to others.

**Conclusion:**

Surveys on parental satisfaction are essential for the success of a pediatric day center. Apart from the doctors abilities to interact with the parents, other factors, such as a short waiting period, a friendly and helpful staff, as well as appealing premises are essential for a high overall level of satisfaction.

## Background

Patient satisfaction, or parent satisfaction in the case of children under medical care, is a construct from social research, which describes the satisfaction of patients with demanded services from the health care system. The National Center for Health services Research and health care Technology Assessment has identified patient satisfaction as one of the three major categories of criteria for the evaluation of health care systems [1]. Specifically the term "patient satisfaction" is described by the discrepancy between the quality of the medical care expected from the patient and the perceived quality of medical treatment. The special aspect of the concept of patient satisfaction is that quality standards are not evaluated any more by teams of experts but by the patients themselves [2].

A very useful tool for the assessment of parent satisfaction is a questionnaire-based survey. The advantages of a questionnaire are manifold: The parents can answer questions anonymously and don't have to fear negative consequences because of their judgment. Questionnaires are, in contrast to interviews, more economic, faster and are regarded as more voluntary from the parents [3-5].

But why is parental satisfaction with a pediatric practice so important?

There are mainly 5 reasons:

1. Parental satisfaction can be used as an indicator of quality care

2. For the best possible medical treatment of children it is indispensable that the doctor includes the parents in the treatment regime

3. Compliance with medical regimen

4. Understanding of medical information

5. In times of the free market economy the new critical patient has a free choice of pediatric day centers

Some authors claim that the parents are not in the state of mind to properly assess every aspect of a pediatric practice since they have their sick children to worry about. However, parents are supposed to be rational and capable of making intelligent choices with respect to their children's health, and if they are capable of this, should they not also be capable of questioning and assessing the activities of the staff as well as the physician?

There is evidence that satisfaction with pediatric medical visits is related to parents' compliance with medical regimes, understanding and retention of medical information, and continuity of care [6-10]. However, there are only few multiitem measures of parent satisfaction with the pediatric practice. That is why the members of the "Ped Mind Institute", an institution which concentrates on clinical research in the field of pediatrics, have developed a parental satisfaction questionnaire. The empirical detectable benefit of such a survey is expressed by the number of identified problems, and the interest, as well as the acceptance of the medical staff in the results of the questioning. Our aim is the identification of factors that lead to a high level of parental satisfaction as well as to the stabilization and improvement of the parents' trust in the pediatric practice.

## Patients and Methods

Between October and November 2010, 127 parents of children who were treated at the pediatric practice in Epe (Germany) were asked to fill out the parental satisfaction questionnaire and 67 responded. After the parents had seen the doctor they were asked for 10 to 15 minutes of their time to fill out the questionnaire anonymously. By asking the parents directly on site a high participation was anticipated.

## The parental satisfaction questionnaire

At the beginning, 27 items were generated. The items were constructed on the basis of several sources, including the "Sleep Laboratory for Children Survey, Dresden" [11], studies of parental satisfaction with pediatric medical encounters [7,12] and interviews with parents of pediatric patients. The items were divided into 3 scales. The scales were as follows: "satisfaction concerning the infrastructure and organization" (13 items), "satisfaction concerning the communicative and empathic competence of the doctor as well as the other staff" (9 items) and finally the "result and the overall impression"(5 items). After each scale the parents had the opportunity to write a comment.

In addition 4 questions were asked for those children who are old enough so that their interaction with the doctor could be assessed by their parents. In one of these questions the children themselves were asked to mark their general impression of the pediatric practice on a smiley scale [13]. Furthermore the questionnaire contains 12 questions regarding sociodemographic information of parents and their children as well as situative conditions. The personal questions were put at the end of the questionnaire with the intention of allowing the parents to adjust and feel secure about the anonymity of the survey.

All items employed a four-point Likert scale. An even number of 4 points was used as respondents may avoid using extreme response categories (central tendency bias). The 4 point scale (very satisfied- satisfied- unsatisfied- very unsatisfied) was coded with the numbers 4 to 1 so that the highest number correlates with the highest degree of agreement. Of these items 6 were negatively worded to reduce potential impact of an acquiescence response bias [14]. A pretest was performed by handing the questionnaire to 32 parents who were told to fill out the questionnaire and mark anything that is not clear. Afterwards they were asked if everything was understandable and if they had any other comments. Cronbachs Alpha was 0,805 which is a relatively good value considering it has to be at least over 0,7. Also all means where settled between 3 and 4, which is already a good indicator for the satisfaction of the parents with the pediatric practice.

## Analysis of the questionnaire

All calculations were performed with the statistic program SPSS, version 16.0. Beside the descriptive presentation of the results we compared the means of the parental satisfaction to the specific characteristics of the parents by using the t-test. Furthermore our questionnaire was analyzed regarding situative and sociodemographic different characteristics by using the scores of the 3 scales.

## Results

### Returns

From 127 parents, 67, (52,7%) agreed to fill out the questionnaire. A detailed description of the sample of parents who participated in the survey is listed in Table 1.

**Table 1:** Description of sample of parents

Description of sample	n	%
*Form filler of questionnaire *		

mother	57	93,4

father	4	6,6

*Age of parents*		

under 20 years	0	0

21-30 years	24	38,7

31-40 years	29	46,8

over 40 years	9	14,5

graduation degree of parents		

secondary modern school	4	7,0

middle school	34	59,6

graduation from high school	19	33,4

*Parents with child at the age of*		

*1-5*	44	70,9

*5-10*	10	16,2

*10-15*	8	12,9

*Gender of child*		

male	33	52,4

female	30	47,6

*Parents with indication for child*		

screening	15	25,4

vaccination	10	16,9

acute illness (flu, stomach ache,...)	28	47,5

chronic illness (obstipation, urinary infection)	4	6,8

controls (after operations)	2	3,4

Which child is presented in the practice		

first child	30	47,6

second child	22	34,9

third or further child	11	17,5

Number of times the parents visited the pediatric practice		

never	1	1,6

once	1	1,6

often	59	96,8

*Insurance status of child*		

statuory health insurance	57	90,4

statutory health insurance & private insurance	3	4,8

private health insurance	3	4,8

*Parents with child after visit*		

with therapy	37	74,0

without therapy	13	26,0

### Satisfaction with infrastructure and organization

The results concerning the infrastructure and organization of the pediatric day center can be seen in Table 2.

**Table 2:** Results concerning infrastructure and organization

	EVALUATION			
QUESTION	**very satisfied**-	**satisfied**-	**unsatisfied**-	very unsatisfied
1. Satisfaction with the telephonic availability of the pediatric practice	76.1%	22.4%	1.5%	0%

2. Friendliness of the staff at the reception	91%	7.5%	0%	1,5%

3 a-d. Questions concerning the consulting hours (duration, waiting period, ...)	79%	17%	3%	1%

4 a-d. Questions concerning infrastructure of the practice (parking possibilities, waiting area,...)	55%	29%	13%	3%

5. Satisfaction with waiting time	66%	28%	6%	0%

### Analysis

The outcome of the general impression of the parents depends on many factors. It starts at the very beginning of their visit to the pediatric day center. Significant levels were reached by the questions concerning the atmosphere and décor of the waiting area, the duration of the consultation hours and the waiting time.

If the parents are "satisfied" with the waiting area, their general impressions are rated higher (mean 3,47) and even significantly (p = 0,036) more higher if they are "very satisfied" (mean 3,76) with the waiting area. Parents who were "very satisfied" with the consulting hours were also more satisfied (mean 3,71) with the practice in general, than parents who were only "satisfied" (mean 3,30). These values reached a significant level in a t-test (p = 0,03).

The overall waiting time was 13,74 minutes and although this is a good value, the mean satisfaction was only 3,6. Three parents came without an appointment, but they did not have to wait longer than others, in fact their mean waiting time was only 6 minutes. We also detected a strong correlation between the length of the waiting time and the satisfaction of the waiting time. The satisfaction of the waiting time of parents who waited up to 10 minutes was 3,86 while the mean satisfaction of parents who waited more than 10 minutes was 3,32. These values are very significant in a t-test (p = 0,004). Furthermore we discovered that parents who were "satisfied" with the waiting time were also mostly "satisfied" with the practice. These values reached a significant value in a t-test (p = 0,02).

### Expertise of staff and doctor

The results concerning the expertise, communication skills and empathy of the medical staff as well as the doctor can be seen in Table 3.

**Table 3:** Results concerning expertise of medical staff and doctor

	EVALUATION			
QUESTION	**very satisfied**-	**satisfied**-	**unsatisfied**-	very unsatisfied
1 a-b. Friendliness and helpfulness of medical staff	89%	9%	2%	0%

2 a-g. Expertise, empathy and communication of the doctor	61%	28%	8%	3%

2 h-j. Interaction of the doctor with the child	64%	24%	7%	5%

### Analysis

The expertise of the staff and the doctor and their ability to respond to the parents play an important role in the overall satisfaction of the parents. For the parents the most important factor for their satisfaction was that the doctor had enough time for them. This reached the highest level of satisfaction, namely a mean of 3,94. This value was highly significant (p = <0,001) in a t-test. The parents who were "unsatisfied" by the way their questions and wishes were taken into consideration by the medical staff, rated their general impression of the practice worse (mean 2,5) than parents who were "satisfied" (mean 3,5). The parents who were "very satisfied" by the way they were treated by the staff rated their overall impression of the practice even higher (mean 3,72) which is highly significant (p = 0,001).

It was also very important, that the doctor listened carefully to what they had to say. If they were "unsatisfied" by the way the doctor had listened to them they only reached a mean overall satisfaction of 2,50. In the case that the parents were "satisfied", they reached a significantly (p = 0,007) higher level of general satisfaction (mean 3,23) and even very significantly higher (p = <0,001) was their general satisfaction (mean 3,88) when they were "very satisfied" with the doctors listening abilities. Furthermore a significant positive correlation between the parents estimations, whether they felt understood by the doctor, have understood the suggested therapy and their general satisfaction could be found.

### Achieved Results

**Figure 1:**
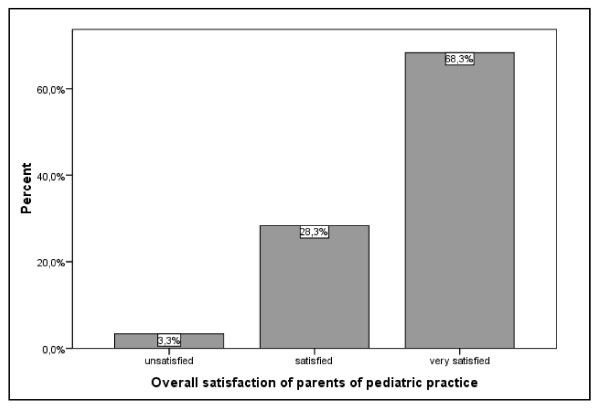
**Overall satisfaction of parents of pediatric practice**.

The achieved results also play an important role for the parental satisfaction. Forty-two parents (70,0%) were "very satisfied" with the achieved results and 66,1% were "very satisfied" with the suggested therapy. 84,2% would definitely recommend the practice to others. Finally the parents were asked what their overall impression of the pediatric practice is and two thirds (68,3%) were very satisfied as can be seen in Figure 1: Overall satisfaction of parents of pediatric practice. In the last question of this part the children who were old enough were asked about their overall impression and 24 children had answered. 83,3% of them "very satisfied" and 16,7% were "satisfied".

Parents who are satisfied with their visit are very likely to come back again. The means of overall satisfaction was 3,86 which is a very high score (max. 4) and therefore 91,5% of the parents would come again with their children. We found a highly significant (p = 0,004) relation between the suggested therapy of the doctor and their general satisfaction. When the parents were "satisfied" with the suggested therapy they only reached a mean general satisfaction of 3,31, but when they were "very satisfied" they reached a mean satisfaction level of 3,81.

We detected no significant relation between the demographic variables tested and satisfaction, except for parents who had a statutory health insurance for their child, who were more satisfied (mean satisfaction 3,7) than parents who had a private insurance for their child (mean satisfaction 3,0). The values reached significance level in a t-test ( p = 0,048).

## Discussion

Vuori stated 1987 that: "Patient satisfaction is an attribute of quality *per se: *without patient satisfaction there cannot be good care" [15]. That is the reason why it is very important, apart from a good professional medical care, that parents of children visiting a pediatric practice are satisfied in general. There are many factors that determine whether the parents are satisfied after their visit to the pediatric day center. Not only is the doctors' ability to communicate adequately, listen carefully or spend sufficient time with the parents essential for a high satisfaction, but also other factors as décor of the waiting area, adequate consultation hours and a short waiting period.

The response rate for mailed questionnaires usually lies at 10-20% [4] and by distributing the questionnaires out personally by the doctor we aimed at influencing the returns rate positively [16]. At least 50 questionnaires should be distributed in order to obtain a representative result [16] and Babbie (1990) stated that a returns rate of at least 50% is adequate for analysis and reporting while a response rate of 60% is good [17]. Therefore our response rate of 52,7% can be valued as adequate. The time of the distribution of the questionnaires is of special importance. The problem with the questioning at the beginning of the visits is that the parents are not able to asses every aspect properly. When handing the questionnaire at the end of the visit it is well-known that the patients have the tendency to judge the practice more favorably (Halo-Effect: the positive experience of the end of the visit retrospectively embellishes the personal recollections)[18], however, it seemed to be the best method. To fully understand the context of our survey, a good knowledge of the German health system, especially for children, is advantageous. In Germany every child is insured either automatically in a family statuory health insurance or in a private insurance. In the case of an illness the first point of contact is the pediatrician, who can decide to transfer the children to a specialist if necessary. All costs are paid by their insurance.

Parent satisfaction with the pediatric day center visit is significantly negatively related to wait times [19]. The mean waiting period was 13,74 minutes, which is a very good value in contrast to the average calculated waiting time of 28 minutes for pediatric day centers [20,21] and therefore we would have expected an even higher rate of satisfaction. A possible explanation could be that the majority of parents (84%) tend to overestimate their waiting time [22] and therefore, a high percepted waiting time is a negative correlation to overall satisfaction [23].

Even more important, and leading to higher levels of satisfaction, is the relationship between the parents and their doctor. We discovered that the highest rate of general satisfaction was achieved, when the parents were "very satisfied" with the time their doctor had given them. This confirms the findings of other authors. Feddock et al. (2010) and Anderson et al. (2007) both discovered that time with the physician was positively related to satisfaction. Furthermore, it even moderated any observed effect of long wait time [19,21]. In a further study Feddock states, that physicians can even mediate the negative effects of long waiting times by spending more time with their patients [24]. For the parents as well as for patients, it is very important that the doctor listens carefully, that they feel understood and furthermore, that they understand the proposed therapy [12,25]. For the doctor on the other hand, a satisfied parent is very important, since there is a significant relation between patients, or, in our case parents', satisfaction and compliance [26]. Then again, a good compliance is essential for a successful treatment [27,28]. We further discovered that, for the parents, the doctor-child relation does not seem very important. Since in a pediatric day center the children are mostly very young, the parents take the role of the primary contact and therefore a good relationship must be built primarily with them.

We discovered that "very satisfied" patients are extremely likely to visit the practice again and all of them would recommend the practice to others. During free market economy times, were the new critical patient or parent has a free choice of pediatric day centers this is of special importance. Other authors also state that satisfied patients will visit the practice again [29]. Furthermore, a satisfied patient will recommend the practice in average on 4 to 5 people [30]. This may not seem much, but 100 satisfied patients will bring the practice 400 to 500 potential new patients.

Finally, we detected no significant relation between the demographic variables tested and satisfaction, except for between the parents who had a statuory health insurance for their child and those, who had a private health insurance. We found that the parents who had a private health insurance for their child were less satisfied in general. One reason could be that they had higher expectations and demands that were not fulfilled. On the other hand, only 3 parents had a private insurance for their child and therefore, the results are more likely not representative. While some authors claim that there is a strong relation between sociodemographic characteristics, such as age, sex, educational background and satisfaction [28,31], other recent findings state that there is a large inconsistency [32]. Francis et al. found no differences in demographic variables and satisfaction [26], Hall and Dornan state in their review that reviewers have failed to reach confident conclusions [33], and Fox and Storms [34] even summarized the situation as follows: "The literature on satisfaction with health care presents contradictory findings about sociodemographic variables...The situation has grown so chaotic that some writers dismiss [sociodemographic] variables as reliable predictors of satisfaction [p. 557]." These results also confirm our findings, of a non-existent relation between sociodemographic variables and parental satisfaction.

There is the argument that patient satisfaction cannot be measured in a way that would generate useful results to improve the quality of care. It is difficult to define what quality means for the parents. There is a large intra-individual variation in what is considered to be qualitatively important and sometimes it can be even ethically unacceptable for the physician of a pediatric practice to satisfy parents' wishes, for example those concerning treatment regimes or other measures [15]. Our aim was to assess, which factors in a pediatric day center lead to a high parental satisfaction level. We have ruled out sociodemographic variables linked to a high satisfaction and found that the ability of the doctor to interact with the parents is the main predictor for a high level of satisfaction. However, also other factors, such as short waiting period, a friendly staff and a nicely decorated waiting area are essential for the smooth running of a practice and as they are rather implied by parents, they turn out negative if not available. We have also received negative news. It was often mentioned that there were not enough toys in the waiting area, for example. As a result we have bought more supplies so that the children have more distractions. We have also found out that the parents were rather unsatisfied when the doctor did not pay attention to their child or did not have enough time in general. The doctor has received a copy of this analysis, and will take these points into account in the future.

## Competing interests

The authors declare that they have no competing interests.

## Authors' contributions

All authors contributed equally and approved the manuscript
